# Braid Entropy of Two-Dimensional Turbulence

**DOI:** 10.1038/srep18564

**Published:** 2015-12-22

**Authors:** Nicolas Francois, Hua Xia, Horst Punzmann, Benjamin Faber, Michael Shats

**Affiliations:** 1 Research School of Physics and Engineering, The Australian National University, Canberra, ACT 0200, Australia; 2 Department of Physics, University of Wisconsin-Madison, Madison, Wisconsin 53706 USA

## Abstract

The evolving shape of material fluid lines in a flow underlies the quantitative prediction of the dissipation and material transport in many industrial and natural processes. However, collecting quantitative data on this dynamics remains an experimental challenge in particular in turbulent flows. Indeed the deformation of a fluid line, induced by its successive stretching and folding, can be difficult to determine because such description ultimately relies on often inaccessible multi-particle information. Here we report laboratory measurements in two-dimensional turbulence that offer an alternative topological viewpoint on this issue. This approach characterizes the dynamics of a *braid* of Lagrangian trajectories through a global measure of their entanglement. The topological length 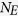 of material fluid lines can be derived from these braids. This length is found to grow exponentially with time, giving access to the braid topological entropy 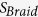. The entropy increases as the square root of the turbulent kinetic energy and is directly related to the single-particle dispersion coefficient. At long times, the probability distribution of 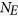 is positively skewed and shows strong exponential tails. Our results suggest that 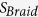 may serve as a measure of the irreversibility of turbulence based on minimal principles and sparse Lagrangian data.

More than a century ago, O. Reynolds showed that watching the dynamics of coloured fluid lines in a flow was a powerful way to uncover the turbulent fabric of the underlying fluid motion^1^. This pioneering study provides a nice illustration that the problem of transport in turbulence is intimately connected to its *Lagrangian* description, the trajectory-based representation of hydrodynamics. Describing and characterizing Lagrangian properties of fluid turbulence is important for a better understanding of many natural and industrial processes, including turbulent mixing, the distribution of plankton in the ocean, or the spreading of pollutants in the atmosphere^2^,^3^. Despite the elegance of Reynolds approach, even now, unravelling the internal fluid motion in natural flows is not a trivial matter because the deformation of fluid lines is usually extremely convoluted^3^,^4^. This observation is not intrinsic to turbulence. Indeed, very complex patterns can be observed when a marker is advected in seemingly simple Stokes flows^5^,^6^, a phenomenon known as chaotic advection^7^. The “chaoticity” of the Lagrangian transport strongly hinders our ability to forecast the consequences of disasters such as volcanic eruptions or pollutant spills on the sea surface. Although basic Lagrangian quantities such as the single particle dispersion offer valuable information, there is a growing realization that multi-particle measurements are instrumental in better describing global transport properties of natural flows^3^,^8^,^9^,^10^.

The merger of ideas from Lagrangian hydrodynamics with those of dynamical systems has been a key route to unraveling the complexity of chaotic advection in *periodic* flows^6^,^11^,^12^,^13^. Crucially, it has been demonstrated that topological features of flows are not abstract mathematical concepts but are an essential part of fluid motion^12^,^13^,^14^,^15^. To date, the application of mathematical tools from topology or dynamical system theory has been largely restricted to idealized maps or simple flow configurations^11^,^12^,^13^,^14^.

Recent advances in laboratory modeling of turbulent flows, the development of experimental particle tracking techniques, as well as the availability of new mathematical methods have made it possible to extend the investigation to non-periodic and turbulent flows^3^,^15^,^16^,^17^,^18^,^19^,^20^,^21^. The combination of particle tracking velocimetry (PTV) and topological tools has recently offered insights into mixing, transition to chaos, and irreversibility in flows^4^,^22^,^23^,^24^,^25^. However, when it comes to measuring key features of Lagrangian transport such as the long-time dynamics of fluid lines in turbulent flows^26^, experimental investigations still encounter numerous problems. Among them is the formidable task of describing the trajectories of many particles that become entangled with a growing complexity. Braid theory and the topology of surface mappings offer interesting means to tackle these questions^6^,^14^,^26^,^27^,^28^. It provides topological tools to measure the entanglement of *braids* made of Lagrangian trajectories. This approach is capable of capturing the deformation of fluid elements using topological considerations and a limited number of Lagrangian trajectories. The method is suitable for studying two-dimensional (2D) flows. So far, the potential of the braid method has been rarely investigated experimentally^5^,^6^,^14^,^29^,^30^,^31^.

Here, we report new experimental measurements of topological braids in 2D turbulent flows. Experiments have been carried out in a broad range of the turbulence kinetic energy by using both electromagnetically forced and Faraday wave driven 2D turbulence. The topological “length” 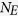 of material fluid lines is derived from the behavior of Lagrangian trajectories, measured using high-resolution PTV techniques. After a transient period, the statistical average of 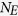 grows exponentially with time and its probability density function (PDF) becomes positively skewed with strong exponential tails. The braid entropy 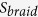 of the flow is measured. We show that 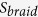 increases as the square root of the turbulent kinetic energy. This study also reveals that 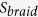 is directly related to the single-particle diffusion coefficient 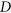. Since quantifying the degree of irreversibility in turbulent flows^32^,^33^,^34^,^35^ is still a matter of active debate, our results suggest that 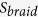 could be a promising alternative measure based on topological considerations and sparse Lagrangian data.

## Results

The experiments are carried out in two different experimental setups used to produce homogeneous two-dimensional (2D) turbulent flows. First, we take advantage of the remarkable similarity between the horizontal motion of particles on the surface of a fluid perturbed by Faraday waves and the fluid motion in 2D turbulence^17^,^18^,^19^,^20^. Though the fluid particle motion has a vertical component, these similarities stem from the ability of Faraday waves to generate lattices of horizontal vortices^17^. These vortices interact with each other and fuel the turbulent motion. In these experiments, the Faraday wave driven turbulence (FWT) is formed on the water surface in a vertically shaken container. The forcing is monochromatic with a frequency set to 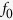. Above a certain vertical acceleration threshold, parametrically forced Faraday waves appear with a dominant frequency of 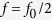 and a wavelength 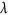. Tracer particles move erratically in the wave field. The forcing scale of the horizontal fluid motion is roughly 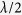. In the second set of experiments, we generate electromagnetically forced turbulence (EMT) in a layer of electrolyte by running an electric current **J** across the fluid cell^36^,^37^. A spatially periodic vertical magnetic field **B** is generated by placing a matrix of magnetic dipoles underneath the cell. The Lorenz **J × B** force produces local vortices at the forcing wave number 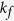 which fuel the turbulent motion. An important aspect of both methods is that energy is injected at an intermediate scale (determined either by the distance between the magnets^37^ or by the oscillon size^17^) in the wave number spectrum, leaving it to the inverse energy cascade to spread energy over a broad range of scales.

### Eulerian energy spectra

**Figure 1:**
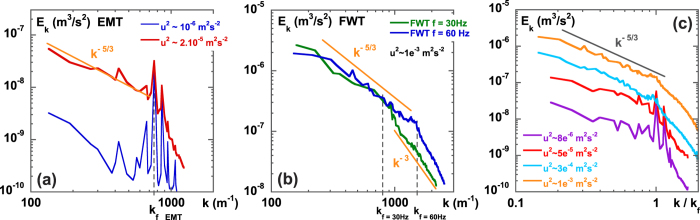
Two-dimensional turbulent flows: kinetic energy spectra measured by PIV. (**a**) Electromagnetically driven flows (EMT): if the flow is weakly forced, forcing scale vortices interact weakly and the spectral energy is localized in a narrow wave number range about 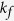. At higher forcing levels, vortices interact in the process of energy cascades and the energy spectrum spreads over a broad range of scales. A continuous Kolmogorov-Kraichnan spectrum is formed that shows a scaling of 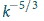 at 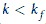. (**b**) Faraday wave driven flows (FWT): Kinetic energy spectra of the horizontal fluid motion. The forcing wave number 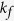 can be changed easily by tuning the forcing frequency 
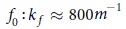
 at 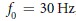, 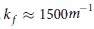 at 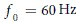. **(c**) Energy spectra versus wave numbers normalized by the forcing wave number 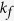.

To visualize the horizontal fluid motion, the liquid-air interface is seeded with 50 μm diameter particles. The *Eulerian* velocity field is measured by using particle image velocimetry (PIV) techniques. Figure 1 shows wave number spectra of the horizontal kinetic energy measured in both experiments for different parameters. The spectral scaling is consistent with the Kolmogorov-Kraichnan prediction of 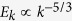 at wave numbers 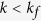, revealing the presence of the inverse energy cascade^38^. At higher wave numbers, 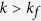, some spectra follow the direct enstrophy cascade scaling 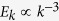, while others are steeper, due to larger dissipation. The use of these two distinct methods allows us to study isotropic 2D turbulence in a broad range of kinetic energies, 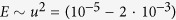 m^2^s^−2^ and forcing scales 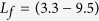 mm.

### Topological braids and topological fluid loops

**Figure 2:**
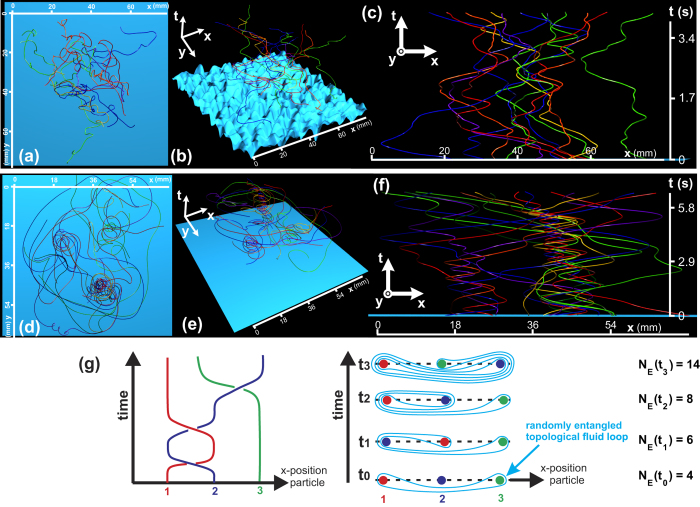
Physical and topological braids in FWT and EMT. Two-dimensional fluid particles trajectories are tracked experimentally by using PTV techniques in the x-y plane for 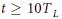 in fully turbulent flows (**a**) driven by Faraday waves 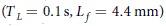 or (**d**) electromagnetically forced 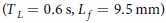. (**b**,**e**), Perspective view of the three-dimensional *x-y-t* strands (time is the third coordinate) built upon the 2D trajectories shown in (**a**,**b**). (**b**) also shows a 3D view of the surface elevation of the disordered Faraday wavefield measured at t = 0 s. (**c**,**f**), the *physical* braids obtained by the projection of the 3D strands onto the *x-t* plane. (**g**) *Left.* Schematics of a *topological* braid made of 3 Lagrangian trajectories. *Right.* Schematics of the temporal evolution of a *topological* fluid loop (blue line) entangled in the same braid. For clarity, the braid is represented as red, blue and green dots at the time of crossing of the 3 particle trajectories. The time evolution of the topological loop “length” 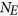 is indicated (see Methods section for computation).

The turbulent fluid motion is also characterized here by using PTV which allows us to measure simultaneously the *Lagrangian* trajectories of hundreds of particles in the horizontal *x-y* plane^17^,^18^. A few examples of the 2D trajectories are shown in Fig. 2(a,d). In these experiments, tracer particles are tracked with high resolution for long times (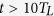, where 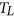 is the measured Lagrangian velocity autocorrelation time). We use tools from braid theory and the topology of surface mappings to characterize, in a topological sense, the deformation with time of fluid elements^6^,^14^,^26^,^27^,^28^,^29^. In the following, we broadly refer to these different tools as the braid description. This method is built upon basic topological considerations and a limited number of Lagrangian trajectories. The connection between Lagrangian trajectories and the topological description of fluid lines is based on two minimal assumptions: i) particles act as local mixers for the surrounding fluid, and ii) fluid lines are impenetrable material objects. In physical terms, it emphasizes that the interaction of a fluid line with the stirring motion of surrounding particles determine completely its temporal evolution.

In this approach, 2D trajectories are viewed as 3D strands, with time *t* being the third coordinate, Fig. 2(b,e). The 3D *x-y-t* trajectories are projected onto the *x-t* (or *y-t*) plane, Fig. 2(c,f). In this plane, trajectories create a *physical* braid made of over- and under-crossings of strands. The crossings are the key topological information upon which the braid description hinges. The crossings of trajectories in 2D turbulence are qualitatively illustrated in Fig. 2(c,f). The braid approach then relies on two distinct objects (Fig. 2(g)):

- the *topological* braid which is really the sequence of crossings of the trajectories previously described.

- the *topological* loop which is like a fluid ribbon entangled within the braid.

The topological braid is based only on the relative position of tracer particles and as such it does not require geometrical information such as the actual distance between strands (Fig. 2(g)_left panel and ref. 14). The topological loop can neither intersect itself nor pass through the braid (Fig. 2(g)_right panel). The degree of entanglement of the loop around the impenetrable strands of the braid can be quantified via a descriptor called the topological “length” 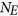 which is also referred to as the *braiding factor* (see details on the computation of 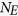 in *Methods*). In the course of time, each crossing along the braid distorts the loop and forces it to stretch or coil around the strands Fig. 2(g). If these deformations are irreversible, the degree of entanglement 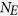 will increase. The time evolution of 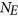 can be computed from the sequence of crossings in a given braid. The topological growth rate 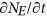 of the loop is expected to capture some features of the behavior of real material lines in a flow^6^,^14^.

### Braid entropy of 2D Turbulence

We measure the temporal evolution of the topological length 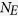 of the fluid loop in FWT and EMT. Measurements are carried out over a broad range of the turbulent kinetic energy of the flow, 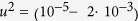 m^2^s^−2^ and for various forcing scales 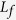. The probability distribution function (PDF) of 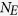 and the statistical mean 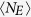 are estimated over at least 10 different braids and up to 100,000 initial topological loops.

**Figure 3:**
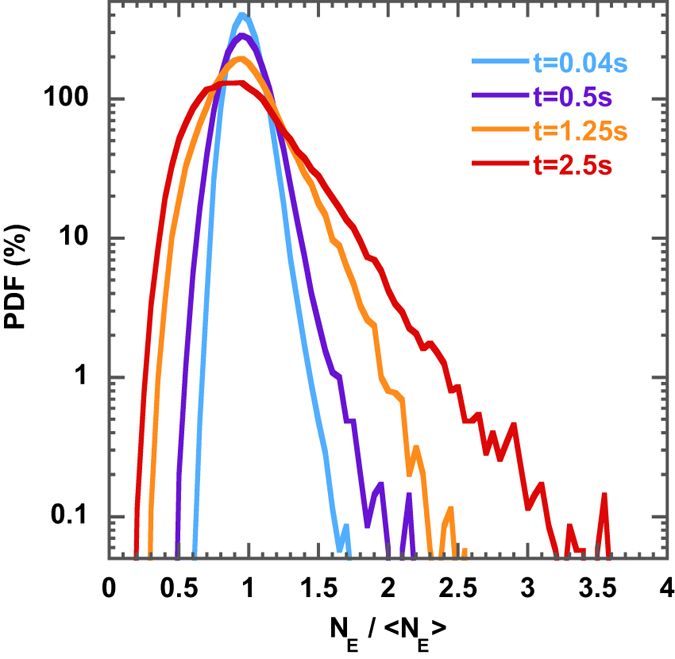
PDF of the topological length *N*_*E*_ of fluid lines in 2D turbulence. Time evolution of the PDFs of *N*_*E*_ normalized by the statistical average 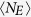 at fixed flow energy 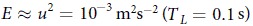. The PDFs are averaged over 15 different braids (made of 80 trajectories) and statistics are collected over 100,000 topological loops.

Figure 3 shows the PDF of 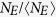 as a function of time at a flow kinetic energy 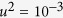 m^2^s^−2^. The initial loop is randomly entangled in the braid; as a consequence, at 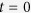 s the PDF is a Gaussian function. After a transient period, the PDF becomes skewed and develops strong exponential tail at large values of 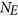. No saturation in the growth of the exponential tail could be observed in the temporal observation window (up to 30 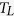 for some runs).

**Figure 4:**
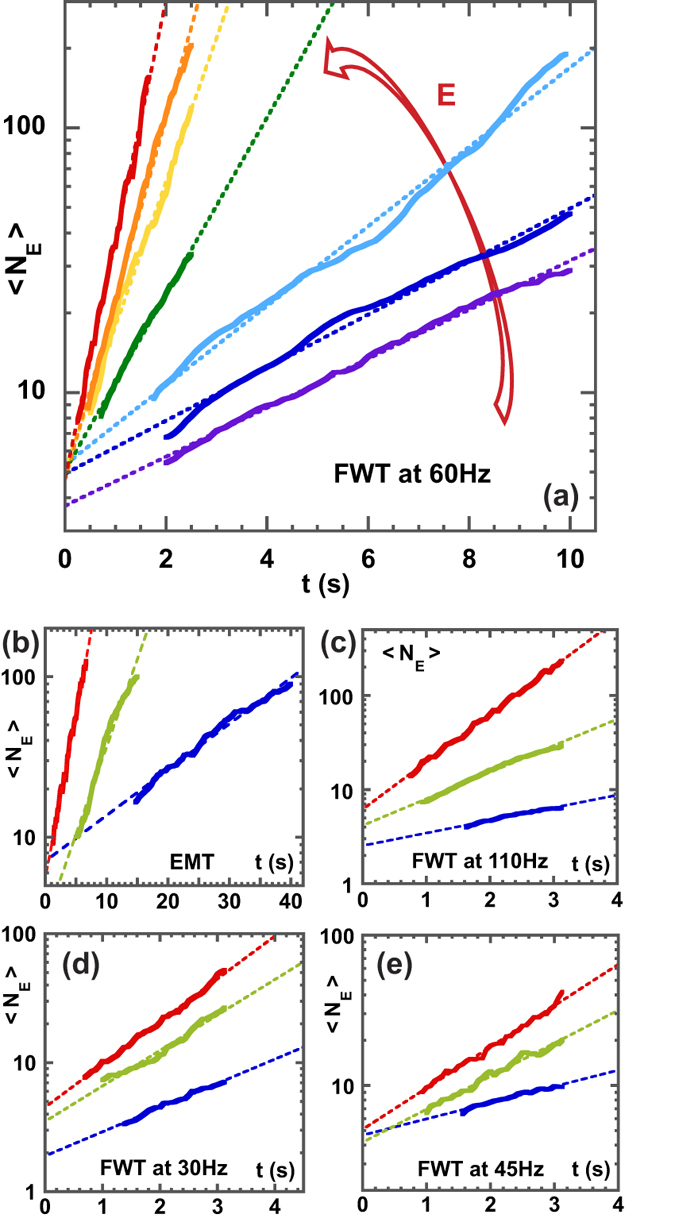
Topological length 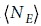 of fluid lines in 2D turbulence. Time evolution of 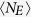 over the range of turbulent kinetic energy of the flow, 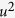 = (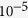 − 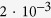) m^2^s^−2^ and for various energy injection scale 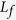. (**a**) FWT at 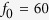 Hz, 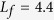 mm, (**b**) EMT at 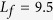 mm, (**c**) FWT at 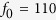 Hz, 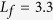 mm, (**d**) FWT at 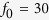 Hz, 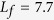 mm, (**e**) FWT at 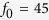 Hz, 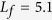 mm. In (**a–c**), 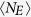 is averaged over at least 15 different braids. Each braid is made of 80 different Lagrangian trajectories. In (**d**,**e**), 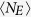 is averaged over at least 10 different braids. Each braid is made of 60 different Lagrangian trajectories. Dashed lines are exponential fits.

Figure 4 shows the temporal evolution of the statistical average 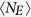 in FWT and EMT as the flow energy is increased. After a transient state, 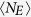 grows exponentially with time and its growth rate increases with the flow energy. This behavior was observed in all our experiments as long as a sufficient number of trajectories compose the braid. The time evolution of 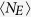 reflects the non-trivial nature of braids made of Lagrangian trajectories in 2D turbulence.

To further characterize this complexity, we measure the braid entropy 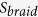 as the growth rate of the logarithm of 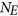 at long times: 
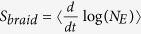
, 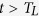. 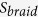 is closely related to the notion of topological entropy^14^. Its definition as the exponential growth rate of topological loops is inherited from the work of Thurston on surface mappings (see ref. 39 and references therein). Basically, 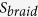 measures the evolution of the number of irreversible deformations that topological loops undergo in the flow.

**Figure 5:**
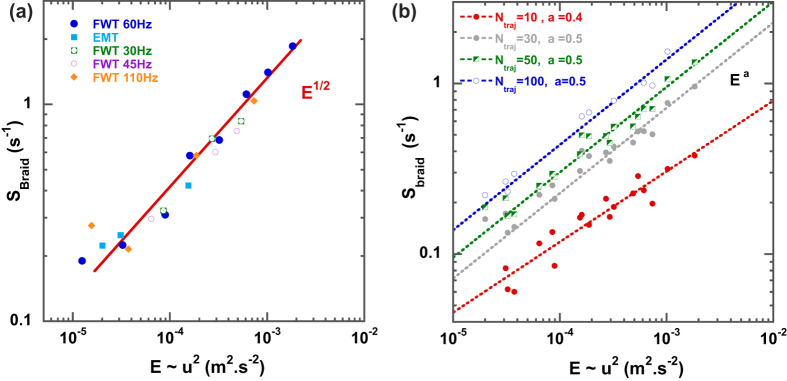
Braid entropy 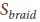 in 2D turbulence. (**a**) The braid entropy 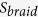 versus the turbulent flow energy 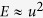, where 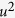 is the mean squared value of the horizontal velocity fluctuations. 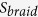 is computed over 10 different braids made of 80 trajectories each. (**b**) 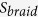 versus 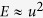 for a varying number 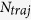 of trajectories that compose the braid. The dashed lines correspond to fit by a power law 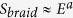.

In these experiments, 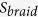 increases as the square root of the flow kinetic energy 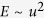, as shown in Fig. 5(a). We observe no appreciable difference between data collected in different experiments, suggesting that 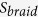 is independent on both the turbulence generation method and on the details of the energy injection. In particular, we detect no dependence of 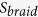 on the energy injection scale 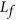. Figure 5(b) shows that the relation 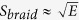 is measured for a number of trajectories 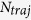 in the braid as low as 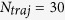.

## Discussion

Recently the concept of chaotic advection was further enriched by considering topological chaos^6^. The characterization of topological chaos hinges on the Thurston-Nielsen classification of surface mappings and on the concept of topological braids^6^,^14^,^26^. Our experimental work concerns topological chaos and explores the potential of the braid description to characterize 2D turbulence. For instance, Fig. 3 shows that the PDF of 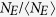 presents growing exponential tails, a feature commonly associated with out-of-equilibrium systems.

The main results of this paper appear in Fig. 5(a), which shows that the braid entropy 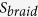 is an increasing function of the flow kinetic energy 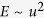, independent of the forcing scale of the turbulent flows. Moreover 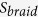 grows as 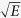 with no sign of saturation. It is expected that the more particles are included in the braid, the better 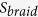 approximates the stretching rate of a “real” fluid line^14^. Batchelor showed that the exponential growth of fluid line in homogeneous turbulence is governed by the deformation of the small fluid elements of which it is comprised^26^. To further test the robustness of our results, we have measured the exponential stretching rate of small fluid elements in our turbulent flows. We have used the finite time Lyapunov exponent method^8^ and found that the average exponential stretching rate 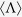 of fluid elements follows: 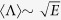, this result^40^ supports strongly the behavior for 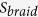 observed in Fig. 5(a). It is quite remarkable that 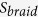 can record the actual behavior of fluid elements from scarce Lagrangian data (in our measurements, as low as 30 trajectories for which the average inter-particle distance is larger than 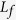), while the computation of the Lyapunov exponents require high spatial resolution measurements of the entire velocity field.

**Figure 6:**
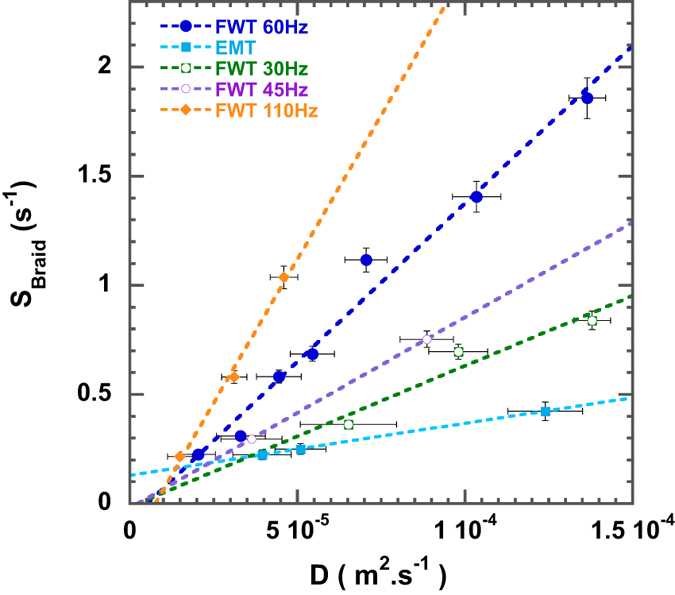
The braid entropy S_braid_ versus the single particle dispersion coefficient *D*. The braids used to compute 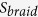 in this graphics are made of 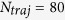 trajectories.

A recent study^41^ reported another type of entropy in 2D turbulent flows, namely the information entropy 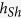. This entropy quantifies the complexity of turbulence in terms of its predictability. Measurements were performed in turbulent flows in soap films; 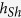 was computed from Eulerian velocity fluctuations. In these experiments, 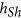 was a decreasing function of 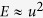. This is in sharp contrast with the behavior of 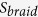 which increases with 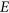 in our work. This discrepancy highlights the fact that the relationship between the Eulerian and Lagrangian descriptions of turbulence remains an outstanding problem^3^,^41^. It also raises questions as to whether there are connections between different types of entropies in turbulent flows^39^,^42^.


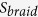
 is a global topological quantity. Although it is connected to transport properties of the underlying flow, its connection to a “metric” descriptor of turbulent transport is not trivial. One of the most basic properties of Lagrangian trajectories is the single-particle dispersion 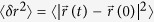 of a particle moving along the trajectory 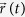. In 2D turbulence, at long times, single-particle dispersion is similar to a Brownian motion, and it reads: 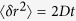 where 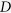 is the diffusion coefficient^18^,^35^. Recent experiments showed that 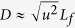 in 2D turbulence, where 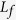 is the energy injection scale of turbulence^18^. The fact that 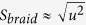 has therefore a remarkable consequence: the braid entropy 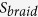 is a linear function of 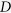 (see Fig. 6). Quantitatively, we have measured: 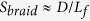. The forcing scale 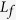 links a single-particle metric characteristic 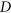, to a multi-particle topological descriptor 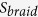.

Although such a connection between single and multi-particle descriptors might sound surprising, it may originate from the uncorrelated motion of the particles that compose the braid. Indeed, we emphasize that the transition to the exponential growth regime of 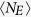 is observed for time scales 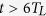 (see Fig. 4). At these time scales, both the single^18^ and pair dispersion computed on the braided trajectories (with inter-particle distance being larger than 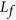) show Brownian statistics. To our knowledge, there is as yet no theoretical understanding as to why the entanglement of independent Brownian trajectories results in an exponential growth of the topological length 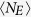. We note that 2D turbulence plays an important role in this phenomenology since the r.m.s velocity 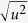 depends on the kinetic energy accumulated in the inertial range. The relation linking 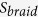 to 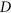 could be useful in oceanography to identify the energy injection scale 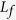 from Lagrangian data^43^.

Much interest lies in determining Lagrangian tenets of turbulence irreversibility that would complement the Kolmogorov energy flux relations formulated in the Eulerian frame^32^,^33^,^34^,^35^. The braid entropy 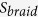 is a promising topological measure of the irreversible deformation of fluid lines in 2D turbulence. On a practical note, the braid approach is particularly suitable for the analysis of natural flows in the ocean for which only sparse data are available. Much work is yet to be done to test the properties and potential applications of the braid entropy in fluid turbulence.

## Methods

### Turbulence generation

In these experiments, turbulence is generated using two different methods. In the first, 2D turbulence is generated electromagnetically in stratified layers of fluid^36^. A 4 mm thick layer of an electrolyte solution (Na_2_SO_4_ water solution, *SG* = 1.03) is placed on top of a 4 mm thick layer of heavier (specific gravity *SG* = 1.8) non-conducting fluid (FC-3283). The fluid cell has a square section of 300 × 300 mm^2^. A matrix of 30 × 30 magnetic dipoles spaced in a checkerboard fashion 10 mm apart is placed under the bottom of the fluid cell producing spatially varying vertical magnetic field 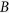. Electric current 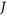 flowing across the cell generates the Lorenz 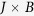 force, which drives 900 horizontal vortices in the top (conducting) layer of fluid^18^,^36^,^37^. The interaction between these vortices, through the inverse energy cascade process, provides the energy that drives the turbulent flow. The bottom layer reduces the bottom drag and makes the flow in the top layer two-dimensional.

In the second setup, Faraday surface waves are used to generate 2D turbulence^19^,^20^. The horizontal fluid motion on the surface of such parametrically excited waves shows strong similarities with the fluid motion in 2D turbulence. In these experiments, Faraday waves are formed in a circular container (178 mm diameter) filled with a liquid whose depth (30 mm) is larger than the wavelength of the perturbation at the surface (deep water approximation). An electrodynamic shaker is used to vertically vibrate the container. The forcing frequency 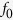 is monochromatic and is set to 30, 45, 60 or 110 Hz. The wavelength 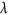 of the sub-harmonic Faraday waves is a function of 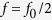. We have recently demonstrated that Faraday waves can generate lattice of horizontal vortices whose characteristic scale is roughly 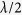. The interaction between these vortices produces a turbulent flow. This method represents a versatile tool of laboratory modeling of 2D turbulence since Faraday wave turbulence can be produced in a broad range of kinetic energy level and forcing scales 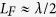, by tuning either the vertical accelerations or the vibration frequency 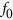.

The use of these two laboratory-modeling methods allows us to study 2D turbulence in a broad range of kinetic energies 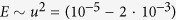 m^2^s^−2^ (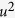 is the mean squared velocity fluctuations) and forcing scales 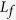 = (3.3–9.5) mm.

### Flow characterization

The flows are visualized by placing 50*μ*m diameter polyamide particles on the fluid surface. The use of surfactant ensures that particles do not aggregate on the surface and it facilitates the homogeneous spatial distribution of the particles. Videos are recorded at high frame rate (60 ~ 600 Hz) and a 16 bit resolution using the Andor Neo sCMOS camera. The flows are characterized using both particle image velocimetry (PIV) and particle tracking velocimetry (PTV) techniques. We use PIV to compute the Eulerian energy spectra of the flows shown in Fig. 1. The PIV velocity fields are computed on a 90 × 90 spatial grid (8 × 8 cm^2^ (FWT), 10 × 10 cm^2^ (EMT) field of view) with a time step of 0.008 s (FWT) or 0.033 s (EMT). The 2D Lagrangian trajectories used in the braid analysis are tracked by PTV techniques using a nearest neighbor algorithm^17^,^18^. In a highly turbulent flow (kinetic energy 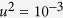 m^2^s^−2^ and integral characteristic timescale 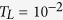 s), hundreds of particles can be tracked simultaneously for 4 s at 120 fps over a 8 × 8 cm^2^ field of view.

### The braid method

#### Topological fluid loops

In the course of time, each crossing along the braid distorts the topological loop and forces it to get more and more entangled in the impenetrable strands of the braid, see (Fig. 2g). It has recently been demonstrated that the level of entanglement of a loop can be described by a quantity 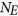 called “topological length” or braiding factor^14^. 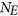 is equal to the number of times the loop crosses a imagined line (horizontal dashed line in Fig. 2(g)_right panel)) passing through all the particles that compose the braid at time 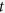. Although it is named topological length, 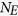 is a topological quantity that ultimately does not require the notion of “distance”. Its time evolution is completely described by the sequence of crossings along a braid.

#### Experimental measurements

To compute the topological braids made of fluid tracers trajectories and their corresponding braiding factor 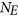, we use tools from the *braidlab* library^14^,^44^ which have been modified to allow the computation to be carried out on a large number of trajectories. The analysis was performed over braids that are composed of 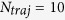 up to 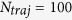 Lagrangian trajectories for which the inter-particle distance is larger than energy injection scale 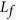. The experimental capacities allow the computation of the single particle diffusion coefficient *D* and the braiding factor 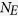 over large statistical samples (~3000 trajectories).

## Additional Information

**How to cite this article**: Francois, N. *et al.* Braid Entropy of Two-Dimensional Turbulence. *Sci. Rep.* **5**, 18564; doi: 10.1038/srep18564 (2015).
